# Morphometric Assessment of Convergent Tool Technology and Function during the Early Middle Palaeolithic: The Case of Payre, France

**DOI:** 10.1371/journal.pone.0155316

**Published:** 2016-05-18

**Authors:** M. Gema Chacón, Florent Détroit, Aude Coudenneau, Marie-Hélène Moncel

**Affiliations:** 1 IPHES, Institut Català de Paleoecologia Humana i Evolució Social, Tarragona, Spain; 2 Àrea de Prehistòria, Universitat Rovira i Virgili (URV), Tarragona, Spain; 3 UMR7194 – HNHP (CNRS – MNHN – UPVD – Sorbonne Universités), Paris, France; 4 LAMPEA, UMR 7269 (AMU – CNRS – MCC – IRD), Aix-en-Provence, France; Universidade do Algarve, PORTUGAL

## Abstract

There appears to be little doubt as to the existence of an intentional technological resolve to produce convergent tools during the Middle Palaeolithic. However, the use of these pieces as pointed tools is still subject to debate: i.e., handheld tool vs. hafted tool. Present-day technological analysis has begun to apply new methodologies in order to quantify shape variability and to decipher the role of the morphology of these pieces in relation to function; for instance, geometric morphometric analyses have recently been applied with successful results. This paper presents a study of this type of analysis on 37 convergent tools from level Ga of Payre site (France), dated to MIS 8–7. These pieces are non-standardized knapping products produced by discoidal and orthogonal core technologies. Moreover, macro-wear studies attest to various activities on diverse materials with no evidence of hafting or projectile use. The aim of this paper is to test the geometric morphometric approach on non-standardized artefacts applying the Elliptical Fourier analysis (EFA) to 3D contours and to assess the potential relationship between size and shape, technology and function. This study is innovative in that it is the first time that this method, considered to be a valuable complement for describing technological and functional attributes, is applied to 3D contours of lithic products. Our results show that this methodology ensures a very good degree of accuracy in describing shape variations of the sharp edges of technologically non-standardized convergent tools. EFA on 3D contours indicates variations in deviations of the outline along the third dimension (i.e., dorso-ventrally) and yields quantitative and insightful information on the actual shape variations of tools. Several statistically significant relationships are found between shape variation and use-wear attributes, though the results emphasize the large variability of the shape of the convergent tools, which, in general, does not show a strong direct association with technological features and function. This is in good agreement with the technological context of this chronological period, characterized by a wide diversity of non-standardized tools adapted to multipurpose functions for varied subsistence activities.

## Introduction

For archaeologists working on the Middle Palaeolithic, it is essential to quantify the variability of artefact shape and test their features by applying a set of parameters, especially to describe and explain variability or standardization in a lithic series in relation to characteristic technological behavioural traditions (e.g., techniques, resharpening, tool functions…).

The technological analysis of lithic assemblages has at times been criticized on account of the use of out-dated methods in comparison to other fields (paleontology and biology in general), and the scant use of quantitative methods [1,2].

The traditional linear dimensions (length, width and thickness) are the standard measurements (following or not the technological axis of the pieces). However the orthogonal dimensions lead to inaccuracies: (1) due to the application of manual dimensions biased by the ambiguities of positioning the piece [3,4], (2) as they reduce the overall tool length, and (3) as they do not plot the geometric configuration of pieces [5].

The rapid development of 3D scanning devices and their application to lithic technology provides a solution for these inaccuracies (e.g. [4,6,7,8,9,10]). 3D methodology offers an automatic and bias-free method for extracting homologous parameters and measurements that are only available with this technology (4) or attributes that were not formerly readily quantifiable [10,11,12,13,14,15,16,17]. 3D scanning records the whole artefact and displays its digitized image as a triangulated point-cloud.

The use of 3D scanning in lithic technology has been adopted in association with the advanced quantitative analysis of data extracted using 3D methods. The main approach is geometric morphometric analysis (GMA), mainly applied in the biological field up until now [18,19,20,21,22]. The major potential application in archaeology relies on the visualization and numerical description of outlines and, therefore, on the use of semi-landmarks and the Elliptic Fourier Analysis (EFA) method [7,23,24,25,26,27,28,29]. In lithic analysis there are far fewer real homologous landmarks than in biology (in the sense of 17). In order to overcome the problem, whole curves (outlines) are used rather than configurations of points, so that lithic morphological features can be studied independently of homological information [23,7]. Lithics are the product of standardized processes of human technological practices that are passed on and sustained by cultural transmission and social learning [30,31]. For this reason, the selection of outlines or discrete points must be related to the distinctive parameters of each artefact and its technical and morphological criteria [23].

The adaptation of EFA to the study of stone tools is far from complete [7], but several works have been published since the first EFA application to lithics [32]. These confirm that this method is relevant as a complementary quantitative approach for the study of human technological behaviour, with a wide array of applications (e.g. resharpening, shape, tool function and variability: [13,23,24,25,26,27,28,33,34,35,36,37,38,39,40,41,42]; technological attributes: [10,12,16,29,43,44,45,46,47]; post-depositional processes: [48]).

Taking into account this research background and the methodological improvements for a better understanding and analysis of the variations of lithic products, this paper focuses on an example of a series of 37 convergent flint tools form the Early Middle Palaeolithic site of Payre, located in the Southeast of France and dated to Marine Isotopic Stage (MIS 8/7) [49,50,51]. It shows that the application of GMA analysis extends and enriches the technological analysis by introducing quantitative data for the comparison of shape, technology and function. The triangular implements of our corpus were produced by discoidal and orthogonal core technology typical of Middle Palaeolithic strategies. The age of the level and the use of these types of technologies explain the presence of non-standardized products, some of which have convergent edges (*déjeté* points, pseudo-Levallois and triangular flakes …). Retouch is limited and does not involve resharpening or the reduction or modification of the point shape. This corpus is consequently a perfect example to apply and test a set of geometric analyses.

The innovative aspects of this paper are (1) a methodological approach combining for the first time EFA 3D analysis with qualitative observations derived from traditional technological and functional analyses and (2) an application to non-standardized and heterogeneous objects from an early lithic assemblage characterized by the high variability of products.

We will attempt to answer the following questions: (1) Can we identify variations and associations between the shape, technology and function of the sampled lithics? (2) Does the third dimension of the contour of lithic artefacts provide informative and meaningful additional data for better analysing patterns of shape variation? (3) Does this methodology work on non-standardized lithic artefacts, as already tested on standardized objects (e.g. bifaces, bifacial and Aterian points…)?

The aim of our research is on the one hand to provide new interdisciplinary data on the status of convergent tools on non-standardized blanks in a given assemblage, and on the other hand to propose a new methodological quantitative approach to study the variability of lithic assemblages. These themes are highly relevant to a better understanding of Early Middle Palaeolithic lithic assemblages.

## The Archaeological Context: Payre Site and Level Ga

**Fig 1:**
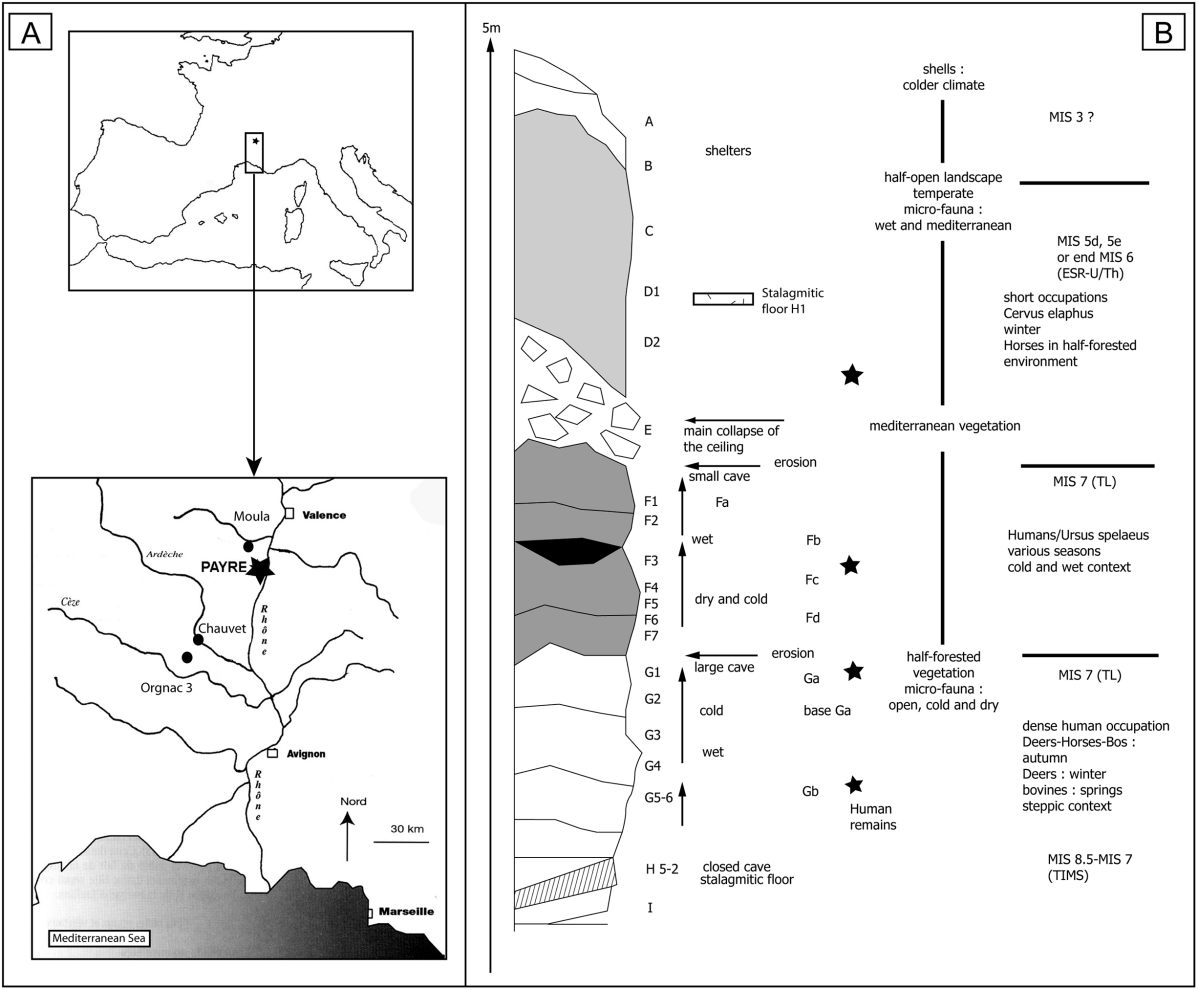
Geographic location (A) and stratigraphy and dates (B) of Payre site [63].

Payre site is located in the Rhone Valley (south-eastern France) (Fig 1A). The site was first a cave, then a shelter before the collapse of the limestone ceiling (Fig 1B). In spite of the varied nature of the site, Neanderthals repeatedly returned at several different periods, perhaps because of its location on a promontory above the Rhone and Payre valleys, providing access to varied environments and resources, especially stone raw materials. Excavations took place between 1990 and 2002 and revealed a 5-m-thick sequence of deposits and eight human occupation levels (Fig 1B). According to ESR, U-Th series, TL and TIMS methods, the sequence is dated to the end of MIS 8 and the beginning of MIS 7 (levels Gb to Fa), and the end of MIS 6 and beginning of MIS 5 (levels E and D) [49,50,52,53] (Fig 1B). Neanderthal remains were discovered throughout the sequence, but most of them are located in the basal levels Gb and Ga [54,55,56,57]. The abundant lithic and faunal assemblages are related to the Early Middle Palaeolithic and are associated with relatively short-term seasonal occupations in a temperate climatic context [50,58,59]. The faunal assemblage shows high species diversity [60,61,62]. Bones were intensively broken, and some were burned. There are indications of fire in each level, and an ashy lens was discovered at the top of level Ga, but no clear hearth structures could be discerned. No spatial organization could be detected in the various Neanderthal occupation phases [50].

Level Ga is one of the richest levels of the site in terms of the quantity of material as well as the technological questions raised by the assemblage. It is 60 cm thick and was excavated over a surface of more than 45 m² (Fig 1B). Excavations revealed a higher density of artefacts here than in other levels (98/m²) and yielded 3,922 artefacts [50]. This layer is a cave deposit with many blocks of various sizes mixed with clay and silt sediments. Post-depositional phenomena include water circulation (breccias) in temperate climatic contexts. The artefact cutting edges are fresh and do not indicate post-depositional disturbance. This layer is a palimpsest of seasonal occupations, as suggested by the faunal remains. Flint artefacts dominate the series (see S1 Table). The flint is from local outcrops and was collected as whole or broken nodules and flakes on the plateau bordering the Rhône Valley, in a 5–15 km perimeter. Some flakes come from further south, up to 60 km from the site [64,65]. Flint pebbles were also collected along the Rhône River. As in the other human occupation levels, flint is associated with local raw materials (basalt, limestone, quartz, quartzite), some of which were worked elsewhere and brought to the site as large tools or large flakes. Cores (flint and quartz) are mainly correlated to discoidal reduction sequences associated with some orthogonal cores [50,66].

## Materials

**Fig 2:**
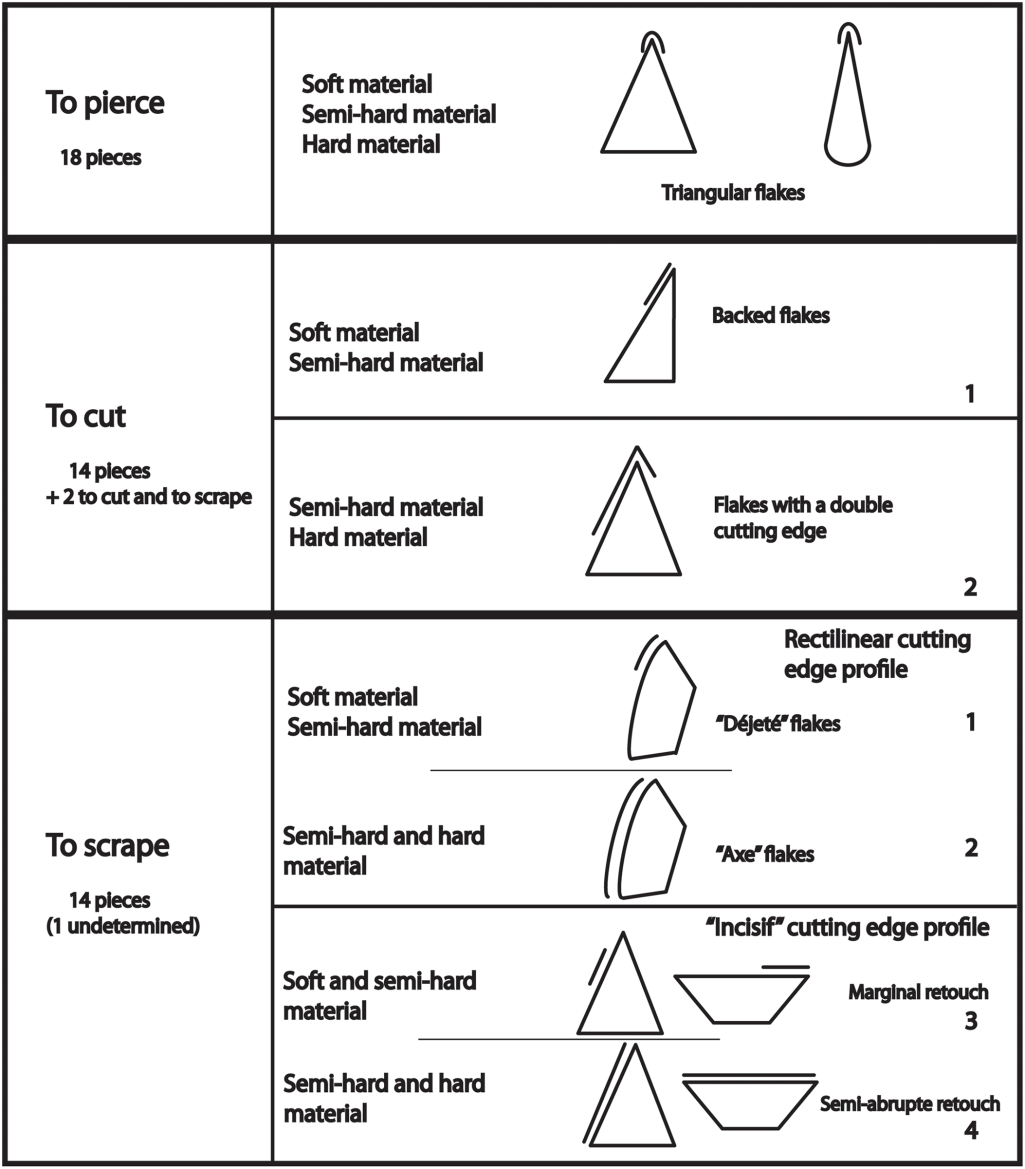
Types of use and location of macro-traces on pointed tools from level Ga [51].

The lithic assemblages of the Middle Palaeolithic levels of Payre total 11 062 pieces (by archaeological levels D = 2216, Fa = 2509, Fb = 813, Fc = 490, Fd = 531, Ga = 3878, Gb = 625) [50]. The convergent and pointed tools of various shapes, sizes and retouch types include 352 pieces (by archaeological levels Ga = 200, Fa = 55, D = 97) [51]. The lithic sample selected for this paper comes from Level Ga and is a part of the previously published corpus [51,63,66,] (Fig 2).

**Fig 3:**
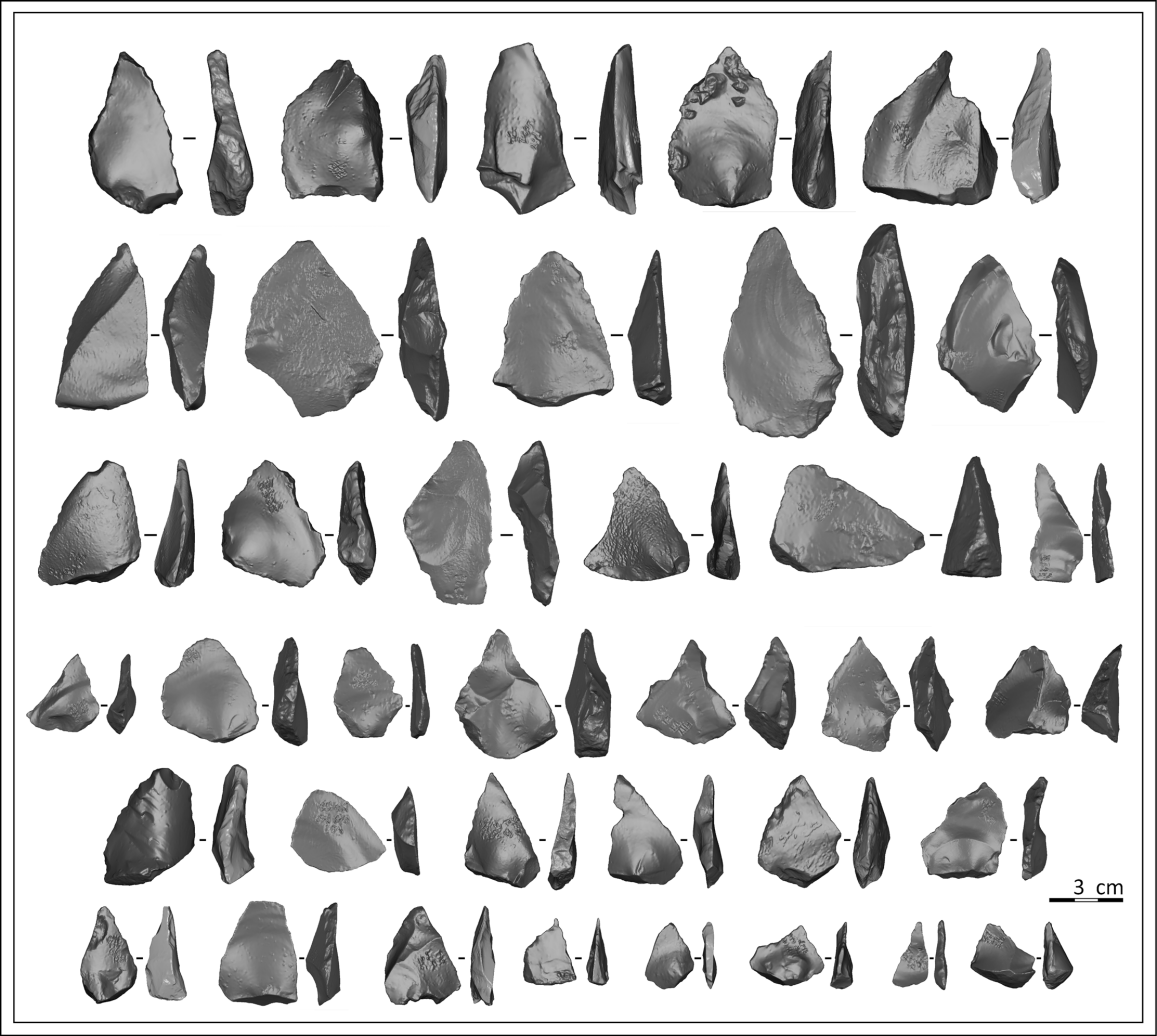
3D models of the convergent tools analysed in this study. The orientation follows the elongated triangular shape defined by the two convergent edges bearing macrowear traces (see Methods section 1).

The thirty-seven convergent tools were selected as they provide wide-ranging data based on technological and typological features as well as macro-wear traces, where available (Fig 3). They are morphologically varied and are produced by typical Middle Palaeolithic knapping methods: discoidal and orthogonal (Quina *débitage* concept) [61], which did not produce standardized flakes (see S1 Table). Only complete convergent tools were taken into account. Most of the flakes are triangular, sub-triangular or trapezoidal-shaped and are commonly identified as asymmetrical *pseudo-Levallois* flakes or backed flakes. They are all made on local flint nodules [64,65]. For this study, we deliberately analysed tools made from the same raw material (flint) in order to avoid an additional parameter which could potentially greatly influence the patterns of shape variation of the tools.

Tools were first studied from a morphological and technological point of view [51]. They were oriented according to their morphological axis, measured and described in order to identify their place in the *chaîne opératoire* regarding the following aspects: size, general morphology, edge shape, type of retouch and pointed end (angle, shape). Retouch partially or totally affects one or both convergent edges and in some cases retouch is not clearly intentional. We kept the same orientation for the 3D scan and the EFA analysis (see Methods) as in the previous study [51,67].

Macroscopic use-wear analysis indicates that the corpus displays macrotraces resulting from use for diverse activities related to butchery, mammal carcass processing and also processing starchy plants. No evidence of projectiles has been identified, but possible evidence of thrusting points has been recorded [51,63]. Initial functional results indicate that it is not easy to establish a clear relationship between shape and function, and that these thirty-seven tools were used as hand tools (handheld points used like a knife, see Figs 2, 3 and S2 Table). Following this first study, we decided to test EFA analysis on 3D contours in order to refine our conclusions and explore other more fitting methodologies to assess the association between the shape, technology and function of non-standardized convergent tools.

## Methods

### 1. 3D surface scanning of the artefacts and digitization of the contours

The lithic objects were scanned with a Nextengine 3D ScanStudio HD using the ScanStudio HD software (Fig 3). This captures objects in full colour with multi-laser precision and, in macro mode, with an accuracy of 0.005 inches with a maximum of 400 samples (points) per inch. This scanner is highly portable and offers excellent operability and acquisition accuracy, although difficulties in capturing the fine details of small objects, especially glossy lithic artefacts, are frequently encountered with this type of laser scanner (see for instance [68,69]). However the objective was here to capture the exact shape of the artefacts and not the fine retouch details, which were studied separately using conventional lithic analysis approaches [50,51]. For each piece, the number of capture views was 12 during 360° rotation of the piece placed vertically with the point facing upwards, followed by two times three consecutive views of the point and base, respectively. All the views were scanned in macro position with the HD (high definition) setting. The 18 digitized images of each specimen were exported into.obj file format to be aligned and merged in Geomagic Studio 11. The final 3D meshes were systematically examined for defects or defaults, especially along the sharp lateral edges. In case of defective models, new scans of the objects were carried out, with slight changes to the angles of the capture views.

The total volume of each artefact was also measured to be used as one of the size parameters in the analyses. The finished 3D models were saved as.ply files and imported into Landmark 3.0 (Institute for Data Analysis and Visualization, IDAV, UC Davis, USA), in order to place the landmarks and pseudo-landmarks along the contour, in three dimensions.

For digitization and subsequent analyses, it is important to respect a systematic and homologous orientation of every object so that the landmarks and pseudo-landmarks follow the same order and direction along the contour. The artefacts were not oriented from a technological point of view, but rather following the elongated triangular shape defined by the two convergent edges bearing macrowear traces. Accordingly, pieces were systematically oriented with the two edges converging upwards and the ventral face towards the frontal view. The contour of each piece is defined, in three dimensions, as the sharpest outline of the artefact. It follows the two convergent edges and passes through the pointed end. When portions of the contour are broad and present two possible paths for the contour, the ventral one is systematically chosen since, from a technological point of view, this is the side that refits directly onto the core surface.

**Fig 4:**
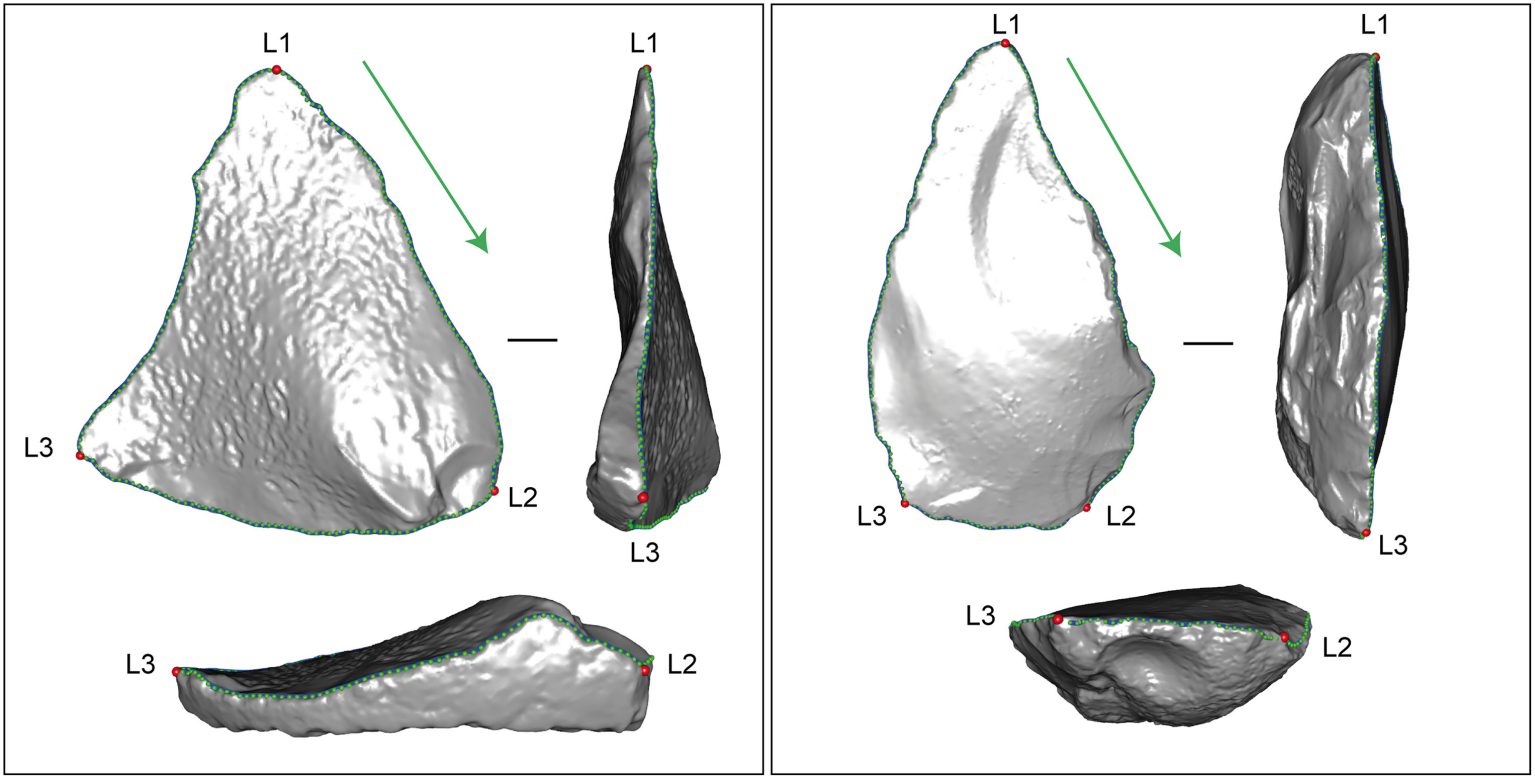
Location of the 3 landmarks (L1, L2, L3) and 196 equally spaced pseudo-landmarks defining the 3D outlines for two pieces with very different outline shapes (not to scale).

A set of three landmarks and 196 pseudo-landmarks were digitized in 3D along the contour of each artefact. The three landmarks are defined as the three angles of the triangular shape of the pieces, with the first landmark being at the “tip of the point”. Landmarks 2 and 3 correspond to each of the maxima of curvature of opposite angles, following a clockwise order on the properly oriented object. 196 equally spaced pseudo-landmarks were also digitized along the contour in a clockwise direction, with landmark 1 as the starting point of the curve (Fig 4 and S1 Dataset).

The 3D Cartesian coordinates of the three landmarks and 196 pseudo-landmarks defining the contour of each of the thirty-seven artefacts were exported in R [70], and the 196 pseudo-landmarks were re-sampled into 196 equally spaced pseudo-landmarks prior to statistical analyses.

### 2. Elliptic Fourier analyses in 3D

The outlines of the artefacts were analysed with Elliptic Fourier Analyses (EFA) in 3D. EFA was originally developed by Giardina&Kuhl [71,72] and has been successfully applied to the analysis of 2D closed outlines of a large variety of biological objects with few or no homologous landmarks (e.g. [73,74,75,76,77]). As noted in the early 1980s by Gero&Mazzullo [32], Fourier analyses are also particularly well suited to analysing contours of non-biological objects such as lithic artefacts. However it is only recently, concomitantly with renewed interest in quantitative size and shape analyses of archaeological objects, that these methods have been more routinely used in lithic studies [7,23,24,25,26,27,28,35,36,44,78]. In the same way as other geometric morphometric methods, EFA results in easier and more rigorous distinction between size and shape differences than other so-called “traditional” morphometric methods [19]. By using inverse Fourier, EFA also offers the opportunity to compute and visualize estimations of mean and extreme shapes in a particular morphospace.

The most frequently used method for the normalization of elliptic Fourier descriptors involves the major axes of the contours [72]. However, the shape of the artefacts analysed here is not characterized by a strong and constant major axis. In order to preserve the orientation of the pieces defined in the analytical design, we use a normalization procedure based on Procrustes superimposition performed only on the three landmarks (e.g. [77,79,80,81]). The 196 pseudo-landmarks are not taken into account in the computation of the Procrustes superimposition, but their aligned coordinates are computed with the normalization parameters calculated for the three landmarks. EFA are then applied (without normalization by the major axis of the first ellipses) on tangent space projections of the Procrustes shape coordinates of the total set of points (3 landmarks and 196 pseudo-landmarks). 3D EFA is a simple extension of EFA in 2D [72,82]. It consists in adding one constant (e0) and two elliptic Fourier coefficients (en and fn) for the third dimension (see for instance [83,84,85]).

The first 12 harmonics (accounting for more than 99% of the cumulative power) were retained for this study. Corresponding Fourier coefficients are analysed first with Principal Component Analysis (PCA) to explore the major trends in shape variation of the artefacts and reduce the dimensionality of the data for subsequent statistical analyses [80]. Several factors (i.e. qualitative variables) obtained from independent macrowear analysis were statistically tested for relationships with shape variation using MANOVAs. discarding factors with insufficient sample sizes for one or several classes (i.e. lower than the number of variables) were discarded, which includes the type of worked material (see S1 Table), and the following factors were selected and tested statistically: localization of macrowear traces, utilization of the point or edges only, and direction of the movement of the utilization action. Depending on available sample sizes of the groups for each test (see S1 Table), the number of variables (i.e. number of first PCA axes) used varied from 5 (82.3% of the outline variance) to 15 (97.5% of the outline variance). Discriminant analyses were also computed to visualize shape variation of the outlines related to those factors. Allometries and relationships between size and utilization of the tools were tested by multivariate regressions and ANOVAs on two different size estimators calculated for each piece: the Centroid Size (CS) and the total volume (see S3 Table).

Procrustes superimpositions, 3D EFA and associated statistics were performed with R (R Development Core Team, 2014), with the “shapes” [86] and “Morpho” [87] packages, and functions written by Claude [83] and Corny&Détroit [77]. Extreme shapes used to illustrate shape variations along axes were computed following the procedure explained in Monti et al. [88].

## Results

### 1. Overall shape variation of convergent tools from Payre: Principal Component Analysis

**Fig 5:**
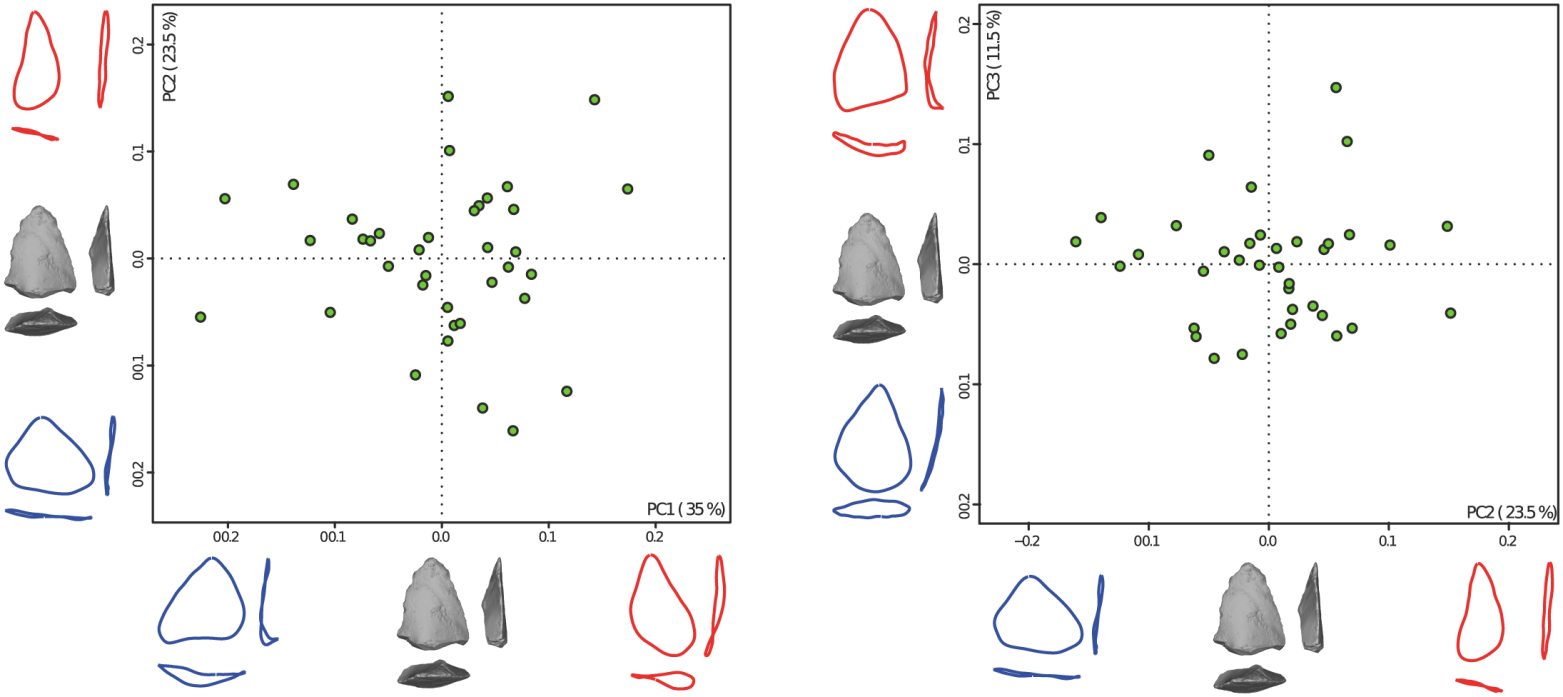
Principal Component Analysis of Fourier coefficients: scatter plots of PC1 versus PC2 and PC2 versus PC3, and visualization of outline shape differences along the PCs.

The PCA of the Fourier coefficients displays rather wide variation in the shape of the 3D outlines of the 37 convergent tools from level Ga from Payre. This variation is mainly distributed among the first principal components (PCs). The first twelve PCs account for slightly more than 95% of the total variance and the first three PCs account for 70% (Table 1). However, the observation of shape variations in the 3D outlines along the PCs shows that the first three axes represent the most significant trends regarding the shape of the convergent tools (Fig 5). The major axis of shape variation (PC1, 35% of the total variance) for the whole sample corresponds to: (1) the asymmetry of the outline in ventral view, with deviation of the proximal part to the left or to the right; and (2) the curvature of the ventrally convex or concave outline in lateral and distal views. PC2 (23.5% of total variance) corresponds to variations in the general elongation of the tools in ventral view, with invariably straight outlines in lateral and distal views. PC3 (11.5% of total variance) corresponds to:(1) the shape of the proximal border, which is straight or convex in ventral view and (2) the curvature of the ventrally convex or concave outline in lateral and distal views. No particular sub-groups are visible in the scatterplot of the 37 convergent tools from Payre level Ga on PC1 *vs* PC2 and PC2 *vs* PC3. However, very few specimens are plotted in the lower left quadrant of PC1 *vs* PC2 (Fig 5), indicating that proportionally short and broad convergent tools with a proximal part deviated to the left in ventral view and a ventrally deviated outline in lateral view are rare in the sample. In addition, the scatterplot on PC2 *vs* PC3 (Fig 5) indicates that there are few short convergent tools with a convex proximal part in ventral view.

**Table 1:** Principal Component Analysis of Fourier coefficients: eigenvalues and percentages of variance for the first 15 PCs.

PCs	eigenvalue	percentage of variance	cumulative percentage of variance
**PC1**	7.306477e-03	34.97312713	34.97313
**PC2**	4.914521e-03	23.52380964	58.49694
**PC3**	2.394788e-03	11.46287666	69.95981
**PC4**	1.606255e-03	7.68849030	77.64830
**PC5**	9.695184e-04	4.64068943	82.28899
**PC6**	7.540074e-04	3.60912638	85.89812
**PC7**	4.767919e-04	2.28220871	88.18033
**PC8**	4.110410e-04	1.96748567	90.14781
**PC9**	3.480887e-04	1.66615870	91.81397
**PC10**	3.351821e-04	1.60438032	93.41835
**PC11**	2.711390e-04	1.29783197	94.71618
**PC12**	2.051760e-04	0.98209386	95.69828
**PC13**	1.569617e-04	0.75131151	96.44959
**PC14**	1.260492e-04	0.60334640	97.05294
**PC15**	8.912893e-05	0.42662390	97.47956

### 2. Shape variation and type of contact action

**Fig 6:**
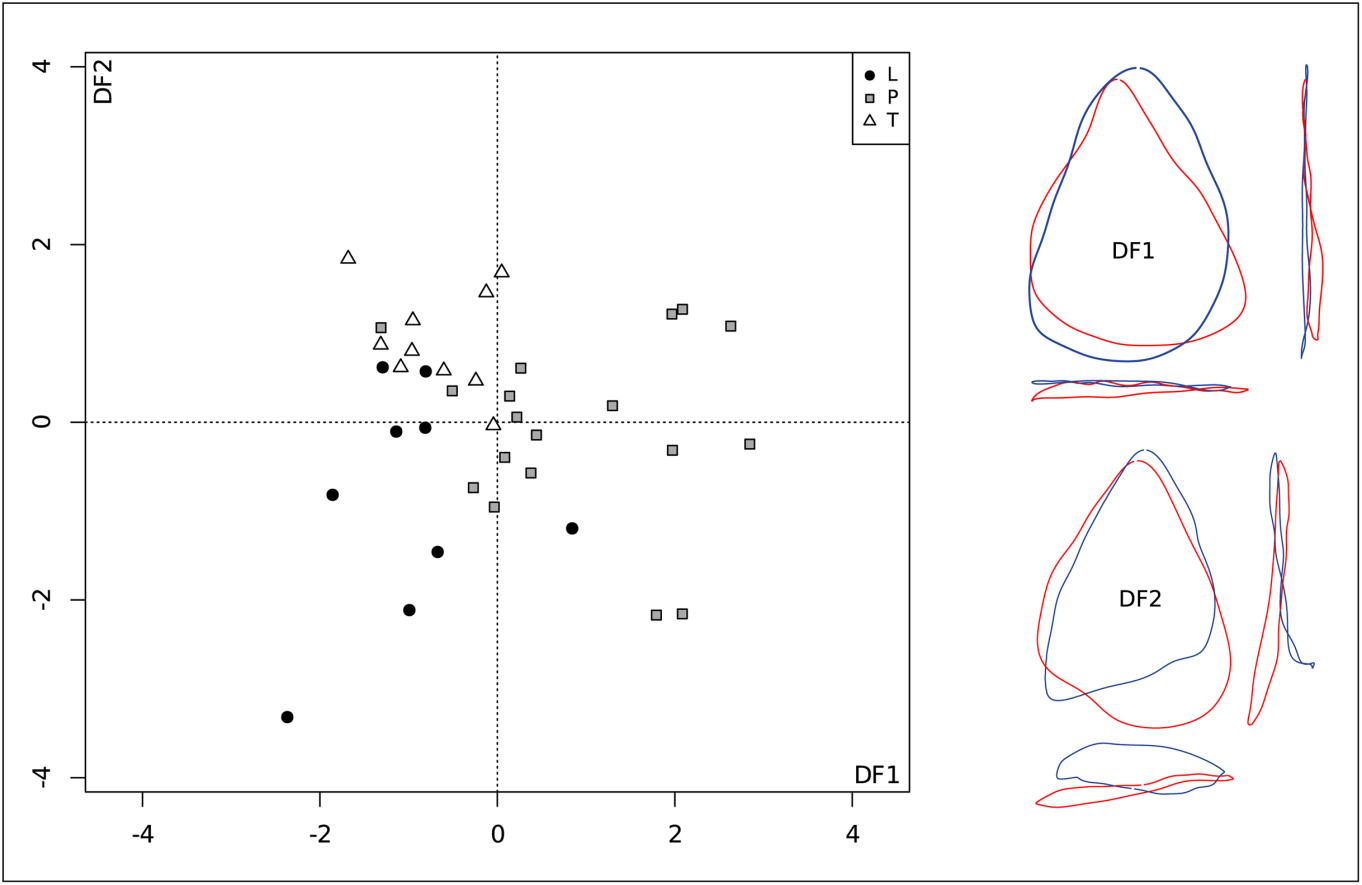
Discriminant analysis for the type of contact action: scatter plot of DF1 versus DF2, and visualization of outline shape differences along the DFs (L = Longitudinal, P = Piercing, T = Transversal).

The MANOVA for the types of contact action (longitudinal, transversal and piercing) [62] (p. 35) computed on the first 9 PCs is significant (Df = 2; F = 1.8956; num/denDf = 18/54; p = 0.037). It shows that there is a statistically significant link between the shape of the convergent tool and the type of use contact action. Overlaps between the three groups of types of contact action are visible on the scatterplot of the discriminant analysis (Fig 6). However, the first discriminant axis tends to separate tools that were used with a contact starting with the point, from tools that were used with longitudinal or transversal contacts. Shape differences along this axis correspond to sharper pointed tools with positive values, as opposed to tools with more rounded tips and more negative values. The second discriminant axis mainly segregates tools used with longitudinal contacts that are plotted towards negative values, from those utilized with a transversal contact towards positive values. Shape variations along this axis show that longitudinal contacts mainly concern asymmetrical tools that tend to present a second “pointed” angle and a wide proximal part, whereas transversal contacts were preferentially made with tools presenting rounded corners, a thin proximal part and a slightly convex outline in lateral view.

### 3. Shape variation and localization of macro use-wear traces

**Fig 7:**
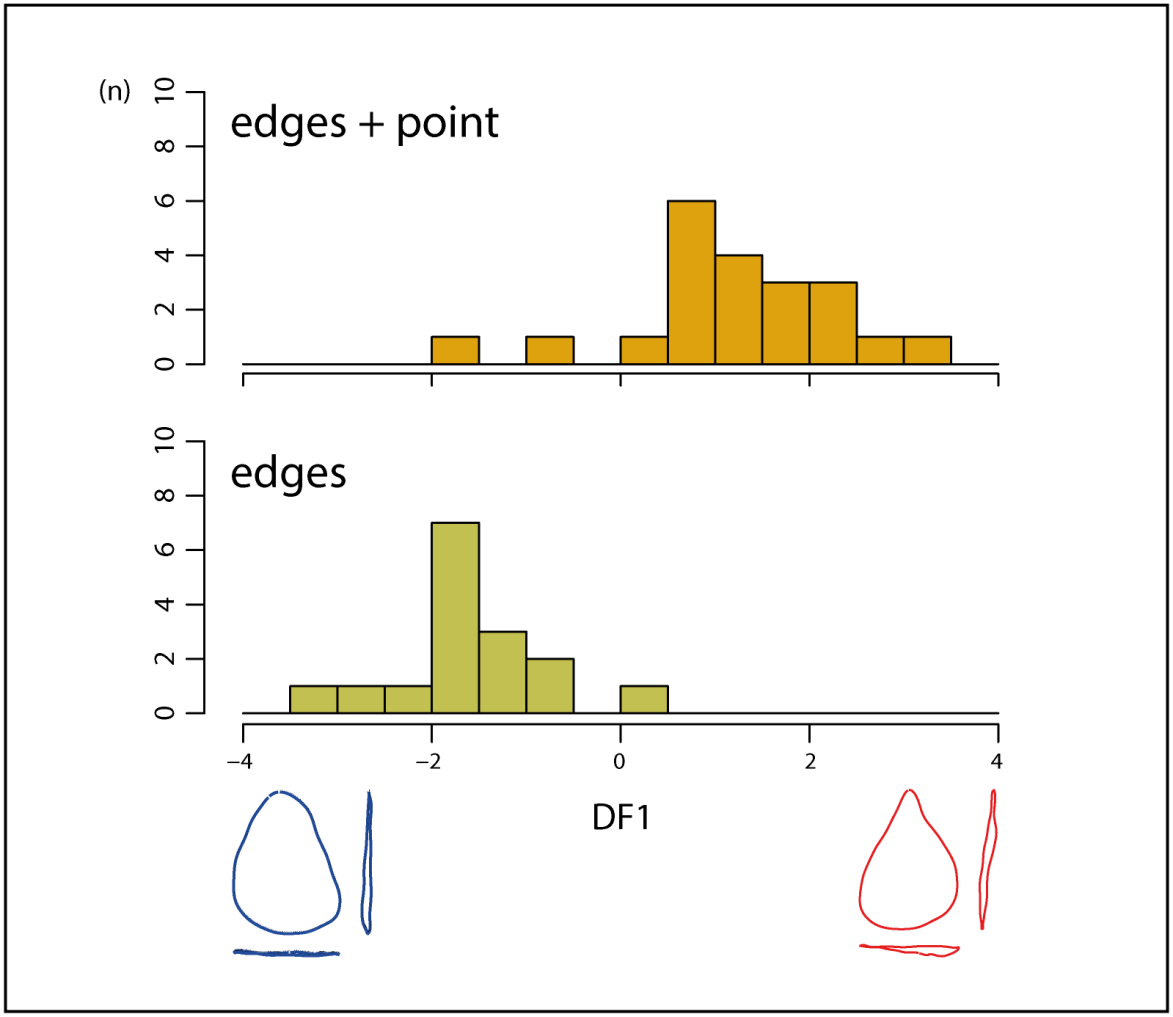
Discriminant analysis for the localization of macro use-wear traces: distribution of convergent tools and visualization of outline shape differences along the DF (edges+points = convergent tools with macro use-wear traces located on the edges and on the pointed distal part, edges = convergent tools with macro use-wear traces located only on the edges).

The MANOVA for the side of the tool where the macro use-wear traces are localized (right, left, both sides) computed on the first 5 PCs is significant (Df = 2; F = 2.0389; num/denDf = 10/62; p = 0.044). However, the scatterplot of the discriminant analysis (not shown here) shows large overlaps between groups, and no clear trends in shape differences along the axes. As the significance of this analysis is probably limited on account of its unbalanced design (very different sample sizes for the three groups), a second test was conducted for the localization of macro use-wear traces. Two groups are considered: the first one consists of convergent tools with traces of use only located on the edges (right and/or left edge), and the second one of tools with traces on the edges and on the pointed distal part. The MANOVA for this factor computed on the first 15 PCs is significant (Df = 1; F = 2.8368; num/denDf = 15/21; p = 0.014). It shows that there is a statistically significant link between the outline shape of the convergent tool and the utilization or not of the pointed distal part. The plot of convergent tools along the discriminant axis shows a rather clear distinction between the two groups (Fig 7). Shape differences along the discriminant axis show that the tools used at the point present a sharper and more elongated tip than the tools only used on the edges.

### 4. Variations in size and shape of the Payre convergent tools

The Centroid Size (logCS, computed from the three landmarks and 196 pseudo-landmarks for each tool) is highly correlated to the cubic root of the volume of the 3D models of the tools (r^2^ = 0.93, p<0.001) (see S3 Table). Although the studied sample includes marked variations in size and volume (Table 2 and Fig 3), it indicates that for this assemblage of convergent tools, there is a strong and constant relationship between the size of the outline and the total volume of the flake.

**Table 2:** Descriptive statistics for size (Centroid Size, in mm) and volume (in mm^3^) parameters (see S3 Table).

	n	Mean	Standard deviation	Minimum	Maximum
**Centroid Size**	37	270.92	77.16	113.96	437.90
**volume**	37	9407.60	7759.14	306.81	34012.32

Despite wide size variations in the total sample, all the statistical tests of shape against size and volume variation (ANOVA and multivariate regressions) yielded non-significant results. In this sample of 37 convergent tools, there is no general relationship between size and outline shape (i.e. no “allometry”), and no significant relationship between tool size and type of use.

## Discussion

The majority of studies applying EFA to lithic technology and function are carried out on standardized artefacts with simpler homologous points in relation to the symmetry of the tools and on 2D contours (e.g. [7,23,24,25,26,33,34,35,36,37,42,44,46]).

This paper is one of the rare analyses of non-standardized artefacts [27,28,29] and the first one to apply 3D EFA to lithic contours to describe the association between the shape, technology and function attributes of a corpus.

This work contributes to validating and widening the use of 3D EFA in lithic technology studies. The obtained results show that this methodological approach is appropriate for analysing and describing three-dimensional shape variations of the edges of convergent tools. These non-standardized lithic tools present no symmetry-related restrictions and the problem of the choice of landmarks and pseudo-landmark positions on artefacts was solved by using a combination of geometric and technological criteria. Indeed, artefacts are the result of repetitive and standardized human technological practices, transmitted and sustained by cultural transmission and imitation [30]. Thus, the selection of outlines or discrete points must be related not only to research questions but also to the specific topological features of each artefact, as well as technical and morphological criteria [23, 45]. In this work, we followed these propositions by placing the landmarks and pseudo-landmarks according to the topological (convergent pieces with three major angles) and technological (orientation) attributes of the piece in order to obtain the actual 3D outline. Those quantified proxies describing the shape of the outlines in 3D were analysed using 3D EFA to explore the actual variability of such artefacts and tested against several qualitative variables derived from independent technological and macro use-wear analyses.

As far as the general shape variation of Payre convergent tools is concerned, there is no clear or direct relationship between technological features and shape variation that can be directly linked to different functions. The PCA of the Fourier coefficients does not identify groups or particular associations of pieces (Table 1, Figs 3 and 5). We observe however that compared to elongated tools, the short convergent pieces used by Neanderthals at Payre almost never present ventrally deviated outlines in lateral views, nor convex proximal parts in ventral views. From a technological point of view, the short pieces chosen for use in different activities have rectilinear ventral faces and are not *déjeté* flakes (see Results 1). This could be related to more efficient grasping precision using exclusively the fingertips when employing small-sized pieces [89,90,91].

Although there is no clear trend or pattern in overall shape variation, our results show several statistically significant relationships between the shape and the use of convergent tools.

The MANOVA for the type of contact actions shows a statistically significant link for the shape of convergent tools (see Fig 6 and Results 2). DF1 clearly separates the pieces used with contact point actions (piercing—red outlines) with a pointed distal part from tools used with longitudinal (cutting) or transversal (scraping) contacts (blue outlines), with more convex edges and distal zones. DF2 distinguishes asymmetrical tools with a wide proximal part used for longitudinal contact actions from pieces with rounded angles and thin proximal parts for transversal contact actions. From a technological point of view, the outlines of these pieces show on one hand backed pieces (red), and on the other hand pieces with a large butt (blue) (Fig 6). These results show the preferential choice of certain shapes in relation to the type of movement and this could be linked to the type of grip used in order to render the piece more comfortable in the hand, either to cut or to scrape, while applying the necessary force [92,93].

The results of the MANOVA for the total localization pattern of the macro use-wear traces are not statistically significant. However, for the second test, if we only consider convergent tools with traces on the edges and tools with traces on the edges and on the pointed distal part, there are significant shape differences between these two groups (see Fig 7 and Results 3). Tools with no evidence of the use of the tip are less pointed. Conversely, pieces showing evidence of tip use are predominantly very sharp pieces. We have to consider that the sharp tip is perfect as an apex to start using the cutting edge (cutting actions and even scraping) whereas other tip morphologies are not suitable for this type of action. These results can be linked to those detailed above concerning the type of contact actions. Both analyses show a clear separation of pieces with pointed shapes and thinner lateral outlines from the more rounded and convex ones, with larger butts.

Lastly, the analysis of size and volume variations shows that, although there is a wide size variation range in the total sample, there is no statistically significant relationship between size variation and outline shape variation nor between the size of the tools and the type of contact actions they were used for (see Table 2, Fig 3 and Results 4).

These results point to the hypothesis that the Neanderthals occupying Payre site during the Early Middle Palaeolithic (MIS 8–7) used non-standardized triangular implements as multi-task tools, with preferential choice of certain shapes depending on the intended contact actions.

Convergent tools (triangular implements or flakes) are ubiquitous in the majority of Middle Palaeolithic lithic assemblages. They are common elements of the Neanderthal lithic toolkit. In the case of convergent tools from Level Ga of Payre, our sample consists of non-standardized Middle Palaeolithic points obtained by flake production, used mainly as butchery knives (see Materials) [51]. They are not standardized Levallois objects produced using a controlled technique [94], as found from MIS 7 onwards in Europe [95], with clear functionality as observed later during this period (e.g. [67,96,97]). Resharpening is not attested and pieces show the same patterns, regardless of whether or not they are retouched. Points were retouched once and rarely thinned. Knappers took advantage of the natural morphology of the pieces produced by discoid and orthogonal core technology, yielding blanks with two convergent edges [46,61]. These core technologies are considered as expedient *débitage* whereas Levallois technology produces standardized flakes which were generally used without retouch [67,94,95,96,97].

Our corpus attests that Neanderthals used all types of knapping products for various activities without major modification and the convergent edges were one of the main selection criteria if we consider our sample of pieces [51,63]. Even if the convergent tools studied are versatile and multi-task tools used for multiple functions, Neanderthals looked for pointed pieces with dihedral and trihedral convergent edges to conduct their activities (retouched or not). They chose pieces with two convergent edges and with shapes facilitating gripping to carry out subsistence-related activities (butchery, mammal carcass processing and processing starchy plants) [51,58,62,63].

Our study confirms that, from methodological and analytical points of view, the use of 3D contour analyses greatly enhances the description and quantification of lithic artefact shape variation. It thus opens new prospects for future research for improving the quantification and reinforcing our understanding of the variability of tools produced and used by Neanderthals, providing new data on the status of convergent tools in the Early European Middle Palaeolithic.

## Conclusion

This study proposes that EFA and landmark/semi-landmark-based methods generate very insightful quantitative information concerning shape variation in lithic artefacts for exploring morphological variation patterns in technologically coherent series. This methodological approach includes many applications that go beyond shape analyses of lithic artefacts per se when combined with quantitative or qualitative variables derived from other more routine lithic analyses. In this case study, 3D shape variables are tested against qualitative variables, which correspond to the technological and functional attributes of convergent tools manufactured by Neanderthal occupants of Payre during the Early Middle Palaeolithic (MIS 8–7).

This work has important implications for the improvement and enrichment of technological lithic studies through the use of quantified, more objective and statistically verifiable observations. These observations represent a significant complement to the qualitative observations derived from traditional technological and functional analyses.

We emphasize in this work the utility of expanding the application of methods and analyses based on 3D scanning in combination with, rather than instead of, more traditional quantitative and qualitative methods [12]. We anticipate that such combinations of quantitative and qualitative analytical approaches will increase the scope of lithic studies and their contribution to our knowledge and understanding of the variability of technological behaviours of past hominin populations, as well as their cultural and behavioural adaptive processes [7,34,42]. In this case, our work provided new data to understand the status of convergent tools on non-standardized blanks during the Middle Palaeolithic showing a privileged choice of certain tool shapes depending on the activities and the type of contact actions.

## Supporting Information

S1 Dataset Raw coordinates of landmarks and semi-landmarks digitized on the 37 pieces analysed in this study. Each row corresponds to one piece (identified by its ID), columns contain Cartesian coordinates. Lcoord 1 to 9 correspond to the X, Y, Z coordinates of landmark 1, 2 and 3; slcoord 1 to 588 correspond to the X, Y, Z coordinates of the 196 semi-landmarks, as digitized on the 3D models (semi-landmark 1 is equal to landmark 1).
(XLSX)

S1 Table Lithic assemblage from level Ga by raw materials and technological categories (values in brackets are percentages, (*) n = 44 are micro-fragments in quartzite) from Baena, J., Moncel, M-H., Cuartero, F., Chacón M.G., Rubio, D. Late Middle Pleistocene genesis of Neanderthal technology in Western Europe: The case of Payre site (south-east France), Quaternary International (2014), http://dx.doi.org/10.1016/j.quaint.2014.08.031.
(DOC)

S2 Table Results of macrowear traces analysis for the 37 convergent tools (SH = semi-hard materials, H = hard materials, IND = indeterminate material; R = right edge, L = left edge, LR = left&right edges; T = transversal, L = longitudinal, P = piercing). (DOC)

S3 Table Centroid size and Volume for the 37 convergent tools. (DOC)
