# Supplementary material for: Morphometric Assessment of Convergent Tool Technology and Function during the Early Middle Palaeolithic: The Case of Payre, France
Source: PLoS One. 2016 May 18;11(5):e0155316. doi: 10.1371/journal.pone.0155316 (PMC4871435; doi:10.1371/journal.pone.0155316)
Supplement: S1 Table — Late Middle Pleistocene genesis of Neanderthal technology in Western Europe: The case of Payre site (south-east France), Quaternary International (2014), http://dx.doi.org/10.1016/j.quaint.2014.08.031. (DOC) [file pone.0155316.s002.doc]

**Supporting Information**

**S1 Table.** Lithic assemblage from level Ga by raw materials and technological categories (values in brackets are percentages, (*) n= 44 are micro-fragments in quartzite) from Baena, J., Moncel, M-H., Cuartero, F., Chacón M.G., Rubio, D. Late Middle Pleistocene genesis of Neanderthal technology in Western Europe: The case of Payre site (south-east France), Quaternary International (2014), <http://dx.doi.org/10.1016/j.quaint.2014.08.031>

|  | **Nodules** | **Whole**  **pebbles** | **Broken**  **pebbles** | **Pebble-**  **tools** | **Cores** | **Flakes**  **& chunks** | **TOTAL** |
| --- | --- | --- | --- | --- | --- | --- | --- |
| **Basalt** |  | 50  (16.78) | 37  (12.42) | 38  (12.75) |  | 173  (58.05) | 298  (7.6) |
| **Quartz** |  | 2  (1.09) |  |  | 6  (3.26) | 176(*)  (95.65) | 184  (4.69) |
| **Limestone** |  | 1  (6.67) | 3  (20) |  |  | 11  (73.33) | 15  (.38) |
| **Quartzite** |  |  | 1  (2.08) | 3  (6.25) |  | 44  (91.67) | 48  (1.22) |
| **Sandstone** |  |  |  |  |  | 1  (100) | 1  (.03) |
| **Flint** | 1  (.03) |  |  |  | 90  (2.67) | 3285  (97.3) | 3376  (86.08) |
| **TOTAL** | 1  (.03) | 53  (1.35) | 41  (1.05) | 41  (1.05) | 96  (2.45) | 3690  (94.08) | **3922** |
