# Supplementary material for: Morphometric Assessment of Convergent Tool Technology and Function during the Early Middle Palaeolithic: The Case of Payre, France
Source: PLoS One. 2016 May 18;11(5):e0155316. doi: 10.1371/journal.pone.0155316 (PMC4871435; doi:10.1371/journal.pone.0155316)
Supplement: S2 Table — (DOC) [file pone.0155316.s003.doc]

**Supporting Information**

**S2 Table.** Results of macrowear traces analysis for the 37 convergent tools (SH= semi-hard materials, H=hard materials, IND=indeterminate material; R= right edge, L=left edge, LR=left&right edges; T= transversal, L=longitudinal, P= piercing).

| **Label** | | **Type of worked material** | **Localisation of macrowear traces** | **Use of the convergent tools** | | **Direction of the movements of use action** |
| --- | --- | --- | --- | --- | --- | --- |
| **Square** | **Number** |  |  | **on edges (right and/or left edge)** | **on the edges and on the pointed distal part** |  |
| L5 | 1099 | SH | R |  | X | T |
| L5 | 1118 | IND | R | X |  | L |
| L5 | 1134 | SH | L | X |  | L |
| L5 | 1174 | SH | LR |  | X | P |
| L6 | 880 | D | L | X |  | L |
| L6 | 393 | SH | R | X |  | L |
| L6 | 907 | D | LR |  | X | P |
| L6 | 993 | SH | LR |  | X | P |
| L7 | 1119 | IND | LR |  | X | P |
| L7 | 1320 | D | LR |  | X | P |
| M5 | 1231 | IND | LR |  | X | P |
| M6 | 476 | SH | R | X |  | T |
| M6 | 550 | IND | LR |  | X | P |
| M6 | 592 | IND | R | X |  | T |
| M7 | 648 | SH | R |  | X | T |
| M7 | 728 | T | R | X |  | T |
| M7 | 737 | SH | LR |  | X | P |
| M8 | 435 | IND | LR |  | X | P |
| N5 | 473 | D | R |  | X | P |
| N5 | 550 | T | LR |  | X | P |
| N7 | 598 | D | LR |  | X | P |
| N8 | 426 | D | LR |  | X | P |
| N8 | 454 | T | R | X |  | T |
| N9 | 62 | D | LR |  | X | P |
| N9 | 190 | D | R | X |  | T |
| O6 | 122 | D | L | X |  | L |
| O7 | 83 | D | LR |  | X | P |
| O7 | 106 | IND | LR |  | X | P |
| O7 | 130 | D | LR |  | X | P |
| O7 | 355 | D | R | X |  | T |
| O7 | 427 | SH | R | X |  | L |
| O9 | 99 | SH | R |  | X | L |
| O9 | 103 | SH | L | X |  | T |
| P7 | 202 | SH | R | X |  | T |
| P7 | 162 | SH | LR |  | X | P |
| P8 | 13 | SH | R | X |  | L |
| P8 | 176 | SH | R | X |  | L |
