# Supplementary material for: Morphometric Assessment of Convergent Tool Technology and Function during the Early Middle Palaeolithic: The Case of Payre, France
Source: PLoS One. 2016 May 18;11(5):e0155316. doi: 10.1371/journal.pone.0155316 (PMC4871435; doi:10.1371/journal.pone.0155316)
Supplement: S3 Table — (DOC) [file pone.0155316.s004.doc]

**Supporting Information**

**S3 Table.** Centroid size and Volume for the 37 convergent tools.

| **Label** | | **Centroid Size** | **Volume** |
| --- | --- | --- | --- |
| **Square** | **Number** |
| L5 | 1099 | 316,6339 | 14276,79 |
| L5 | 1118 | 342,4404 | 18014,31 |
| L5 | 1134 | 237,5606 | 4765,60 |
| L5 | 1174 | 146,8316 | 1103,77 |
| L6 | 880 | 269,5896 | 8313,18 |
| L6 | 393 | 296,7827 | 10324,73 |
| L6 | 907 | 144,0598 | 934,14 |
| L6 | 993 | 236,3585 | 4273,38 |
| L7 | 1119 | 244,8245 | 5612,98 |
| L7 | 1320 | 371,9323 | 21518,12 |
| M5 | 1231 | 198,0733 | 4866,65 |
| M6 | 476 | 277,2098 | 6018,98 |
| M6 | 550 | 206,5473 | 3523,28 |
| M6 | 592 | 259,2400 | 4764,88 |
| M7 | 648 | 316,1371 | 13490,15 |
| M7 | 728 | 294,7524 | 6334,32 |
| M7 | 737 | 160,0046 | 1384,17 |
| M8 | 435 | 374,4513 | 18888,15 |
| N5 | 473 | 224,6602 | 3875,01 |
| N5 | 550 | 113,9599 | 306,81 |
| N7 | 598 | 252,8325 | 7153,20 |
| N8 | 426 | 437,8993 | 34012,32 |
| N8 | 454 | 377,6681 | 17582,36 |
| N9 | 62 | 188,6987 | 2716,31 |
| N9 | 190 | 272,7745 | 8720,35 |
| O6 | 122 | 213,2065 | 2486,73 |
| O7 | 83 | 279,4545 | 11983,72 |
| O7 | 106 | 246,0093 | 7158,41 |
| O7 | 130 | 265,6230 | 8251,32 |
| O7 | 355 | 335,4555 | 16496,61 |
| O7 | 427 | 252,1163 | 6137,67 |
| O9 | 99 | 285,4314 | 3474,62 |
| O9 | 103 | 197,4854 | 2434,04 |
| P7 | 202 | 420,2102 | 27228,21 |
| P7 | 162 | 368,4176 | 16594,48 |
| P8 | 13 | 245,6755 | 7708,20 |
| P8 | 176 | 353,1641 | 15353,23 |
